# A robust index for metal artifact quantification in computed tomography

**DOI:** 10.1002/acm2.14453

**Published:** 2024-06-25

**Authors:** Jochen Cammin

**Affiliations:** ^1^ Department of Radiation Oncology University of Maryland Baltimore Maryland USA

**Keywords:** artifact index, computed tomography, Gumbel distribution, metal artifacts, quality assurance

## Abstract

**Background:**

Objective assessment of metal artifact strength and the effectiveness of metal artifact reduction algorithms in computed tomography requires a quantitative metric. Metrics described in the literature are typically employed to compare the artifact strength in images reconstructed from the same raw data, but their robustness against varying scan conditions and repeated scans over time as it occurs in periodic quality assurance has not been investigated.

**Purpose:**

A new robust metric for quantifying metal artifacts in computed‐tomography images is proposed and compared to other commonly used metrics.

**Methods:**

The proposed artifact metric is based on the location parameter of the Gumbel distribution, described previously in the literature, but normalized to the location parameter in a background region‐of‐interest to obtain a noise‐independent artifact metric. The metric was compared to three other quantitative metal artifact metrics (artifact‐index, contrast‐to‐noise ratio, Gumbel‐evaluation method) by evaluating metals artifacts in phantom scans and in clinical images. Robustness of the artifact metrics was evaluated using repeated scans with varying noise and against small variations in the selected regions‐of‐interest.

**Results:**

The proposed artifact metric was independent of the underlying image noise and could be reproduced more consistently under slight changes of the region‐of‐interest within the artifact than the other investigated methods. The coefficient‐of‐variation was 5.7% on average with varying regions‐of‐interest in phantom scans and 2.5% in patient scans compared to 9.2% in phantoms scans and 9.9% in patient scans for the next‐best performing noise‐independent metric. Setup reproducibility was better than 5% and was comparable to the other metrics. The new metric correlated linearly with the artifact strength. The contrast‐to‐noise ratio, although often used in artifact quantification, was found to be an inadequate metric due to its lack of robustness against minute changes in the position, size, and pixel values of the region‐of‐interest chosen for calculating the metric and because it showed no correlation with the artifact strength.

**Conclusions:**

A new metal artifact metric has been proposed that is robust under changing scan conditions and less sensitive to user‐dependent choices of the region‐of‐interest than other metrics. The new metric is straightforward to calculate and simple to implement in software commonly used for evaluation of medical imaging systems.

## 1 INTRODUCTION

High‐density materials such as metal implants in patients can cause severe artifacts in computed tomography (CT) images.^1^, ^2^ These metal artifacts adversely impact the diagnostic value of CT images^2^ and reduce the accuracy of structure delineation (“contouring”) and dose calculations in radiation therapy applications.^3^, ^4^ Due to the detrimental effect of metal artifacts on the clinical utility of CT images, numerous methods have been developed over time to reduce or mitigate these artifacts.^5^ To conduct a meaningful and objective investigation of metal artifacts, and to evaluate the effectiveness of metal‐artifact reduction (MAR) algorithms, a quantitative measure of the artifact strength is needed.

While many image quality indices, such as noise, spatial resolution, and contrast resolution, have well‐defined quantitative metrics, there is no consensus in the medical imaging community on how to quantify metal artifacts.^6^ Several authors employed a qualitative visual assessment of the artifact strength by observers.^7^, ^8^ Some authors devised or applied quantitative metrics. An *artifact index* (AI) was used in several studies, based on the standard deviation of pixel values in a region‐of‐interest (ROI) that encompasses the metal artifacts.^9^, ^10^, ^11^ Other authors employed the *contrast‐to‐noise ratio* (CNR).^12^, ^13^, ^14^, ^15^ Imai et al. devised the “*Gumbel evaluation method*” (GEM) for quantifying fine noise streaks in CT images, an approach motivated by extreme‐value theory.^16^, ^17^ Imai's method was adopted by several authors to quantify the strength of metal artifacts in CT images.^18^, ^19^, ^20^

Typically, the artifact strength is measured in different reconstructions of the same raw data, for example, to assess the effectiveness of a MAR algorithm, or to compare several different MAR algorithms. It is straightforward to ensure consistency of the selected ROIs in these situations. The noise magnitude has little influence on the comparison because the evaluation is made relative to the same baseline. However, consistency of the ROIs or even the underlying noise magnitude cannot be guaranteed if the comparison is made between datasets acquired on different CT scanners, or if the scans were acquired at different times on the same scanner.

Each of the beforementioned metrics has strengths and weaknesses that will be demonstrated later in this work: The AI is independent of the noise magnitude but sensitive to the size of the ROIs. The CNR is susceptible to minor changes in average pixel value and standard deviations. The GEM is more robust under ROI variations than AI and CNR but is linearly dependent on the noise magnitude. A metric suitable for quantifying metal artifacts should be reproducible under varying noise conditions and robust against small variations in the chosen ROIs.

In this work, an improved and robust metal artifact index was devised that combines the advantages of other metrics found in the literature without their disadvantages. This new index is termed the *robust artifact index*, or ρ (*rho*)‐index. The ρ‐index was tested for independence from the noise magnitude, robustness under small variations of the selected region‐of‐interest, and correlation with the artifact strength to show that it is a suitable metric for the comparison of metal artifact strength in periodic quality‐assurance measurements and in clinical assessments. In this study, the term ‘noise’ specifically denotes the magnitude of image noise, which is defined as the standard deviation of pixel values.

## 2 METHODS

### 2.1 Summary of existing artifact quantification metrics

**FIGURE 1:**
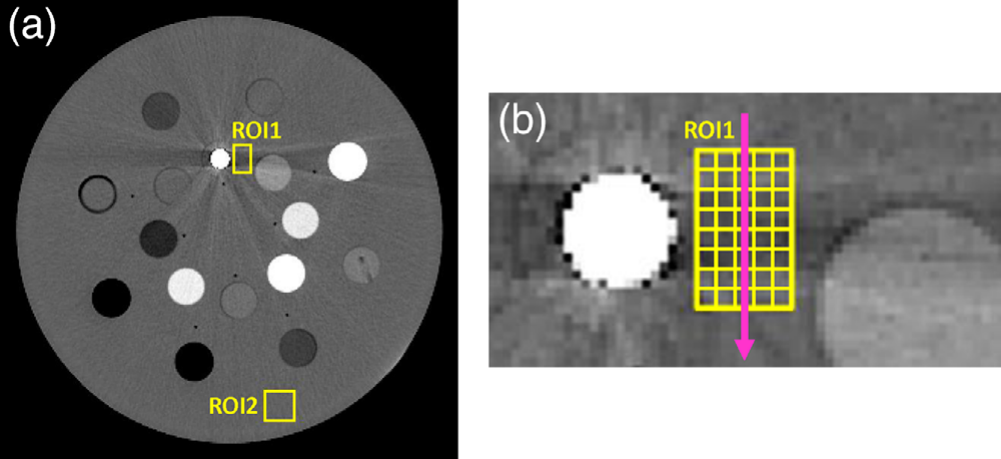
(a) CT image of the electron‐density phantom with a titanium rod to measure the noise dependence of different metrics for metal artifact quantification. ROI1 is located in the artifact region and ROI2 in a background region. (b) Illustration of profile calculation of pixel values perpendicular to the streak direction (vertical arrow).

The AI is calculated from the standard deviation (SD) of pixel values in two ROIs. ROI1 is placed in an area affected by the metal artifact but does not include the metal itself. ROI2 is placed in a homogeneous part of the phantom or patient that is not affected by metal artifacts and has an average pixel value similar to the tissue or material in ROI1 (see Figure 1). The artifact index is defined as $\mathrm{AI}\;=\sqrt{{\mathrm{SD}}_{\mathrm{ROI}1}^{2}\;-\;{\mathrm{SD}}_{\mathrm{ROI}2}^{2}}\;$, and is simply the standard deviation of pixel values in an area affected by metal artifacts with the contribution of regular image noise removed. The CNR metric is calculated from the same two ROIs but in addition, it uses the average (AVG) pixel value in each ROI: CNR $=|{\mathrm{AVG}}_{\mathrm{ROI}1}-{\mathrm{AVG}}_{\mathrm{ROI}2}|/\sqrt{{\mathrm{SD}}_{\mathrm{ROI}1}^{2}\;-\;{\mathrm{SD}}_{\mathrm{ROI}2}^{2}}$. The GEM uses only ROI1 and relates the rank‐ordered list of maxima of adjacent pixel value differences in profiles perpendicular to the artifact streaks to the estimated cumulative probability function of the Gumbel distribution. The strength of the artifact is quantified by the location parameter of the Gumbel distribution obtained from a linear fit to the Gumbel plot.^16^ Due to its design, the GEM is sensitive to image noise.

### 2.2 The robust artifact index (ρ‐index)

The ρ‐index adopts the approach of the GEM but mitigates the noise dependence in the same way as the AI, using a quadratic subtraction of the noise “artifact” strength in the background ROI2. The steps to calculate the ρ‐index are as follows:

1. Define a rectangular ROI1 on the CT image in an area affected by metal artifacts. Do not include the metal implant itself. ROI1 should be orientated so that profiles of the pixel values can be calculated perpendicular to the streaks (see Figure 1). The ROI should be large enough to allow for the calculation of at least five profiles.
2. Calculate profiles *p_i_*, *i *= 1.. *n*, perpendicular to the artifact streaks, where *n* is the number of pixel columns in ROI1.
3. In each profile *p_i_*, calculate the (unsigned) maximum *m_i_* of the differences of adjacent pixel values (PV): $m_{i}=\max_{j}\left|{\mathrm{PV}}_{j+1}^{i}-{\mathrm{PV}}_{j}^{i}\right|\;$, where the index *j *= 1.. *k−*1 runs over the *k* pixels within profile *p_i_*.
4. Sort the set of *m_i_* in ascending order to obtain an ordered list of maxima, ${\tilde{m}}_{i}$.
5. Calculate the estimated cumulative probabilities of the Gumbel distribution $\hat{F}\;\left(i\right)=i/\left(n+1\right)\;$.
6. For each *i *= 1.. *n*, calculate $-\ln(-\ln\hat{F}\left(i\right))\;$ and plot against ${\tilde{m}}_{i}$.
7. Fit the data points [${\tilde{m}}_{i},-\ln\left(-\ln\hat{F}\left(i\right)\right)$] with a straight line y=ax+b.
8. Calculate the location parameter $\lambda_{\mathrm{ROI}1}$ of the Gumbel distribution from the fit parameters as $\lambda_{\mathrm{ROI}1}=\;-b/a$.
9. Repeat steps 2 through 8 for ROI2 placed in a uniform region of the phantom not affected by metal artifacts. The orientation of ROI2 does not matter since there are no streaks.
10. Calculate the ρ‐index as
(1) $$\rho \;=\sqrt{\lambda_{\mathrm{ROI}1}^{2}\;-\;\lambda_{\mathrm{ROI}2}^{2}}\;.$$

The *ρ*‐index has the same unit as the image pixel values, which are typically Hounsfield Units (HU) for CT.

### 2.3 Quadratic sum rule for the Gumbel location parameter

It is shown empirically that the location parameter *λ* of the GEM in a noise‐only ROI differs from the standard deviation σ of pixel values in the same ROI only by a scalar factor *s *: λ=s×σ. This observation implies that Gumbel location parameters can be added in quadrature, just like the noise standard deviation, motivating the noise normalization formula in Equation (1).

Realistic noise‐only images were generated by subtracting images from two consecutive scans of an electron‐density phantom acquired over a wide range of tube currents. The location parameter *λ* of the GEM was calculated for each noise magnitude, *λ* was plotted against the generated noise, and linearity was confirmed with a straight‐line fit to the data.

### 2.4 Comparison of artifact metrics

The robustness of four different artifact metrics (AI, CNR, GEM, and ρ‐index) was compared with respect to noise, choice of the ROIs, setup reproducibility, and varying metal‐artifact shapes in both phantom and patient scans. For the phantom scans, an electron‐density phantom (Model 467, Gammex, Middleton, WI), Figure 1, was scanned in a SOMATOM go.Open Pro CT simulator (Siemens Healthineers, Forchheim, Germany). The phantom contained various tissue‐mimicking materials and metal inserts made from titanium and stainless steel alloys. The data was reconstructed with filtered‐backprojection with and without metal artifact reduction (iMAR, Siemens Healthineers, Forchheim, Germany). Patient scans were obtained from The Cancer Imaging Archive (TCIA) under a Creative Commons License.^21^ One‐tailed paired *t*‐tests were performed to test for statistical significance of robustness differences between the artifact metrics.

#### 2.4.1 Dependence on noise

The metal artifact metrics were calculated using ROI1 and ROI2 in Figure 1(a) and evaluated at different noise magnitudes. The phantom was initially scanned using a high exposure setting to obtain a low‐noise image. Noise‐only images with varying noise magnitudes were then added to the original low‐noise phantom image. These noise‐only images were generated by scanning the phantom under different exposure settings without any metals, and then subtracting two scans. Noise was added artificially instead of scanning the phantom with different exposures to ensure that any variation in the measured artifact metric resulted solely from noise, rather than noise‐independent acquisition or reconstruction effects. This approach mitigates potential variations in artifact patterns, for example, due to different gantry angles at the scan start that cannot be controlled by the user.

#### 2.4.2 Dependence on choice of the ROIs

ROI1 and ROI2 were redrawn five times on each scan and the artifact metrices were recalculated for each ROI variation to assess robustness against changes in the ROI selection. The metrices were evaluated in both MAR and non‐MAR reconstructions for the phantom scans.

#### 2.4.3 Phantom scans

**FIGURE 2:**
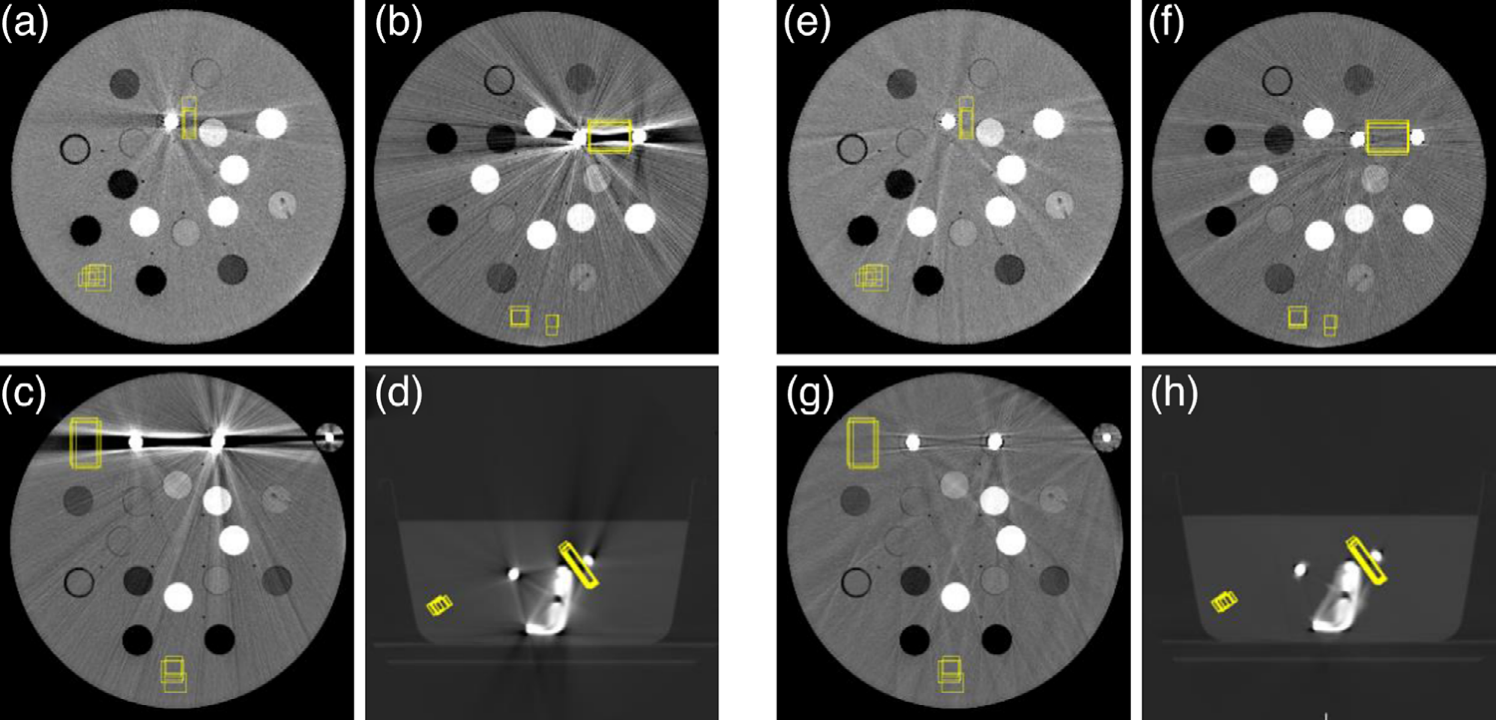
Four examples of metal artifacts in phantom scans. (a)–(b): reconstructions without metal‐artifact reduction. (e)–(h): reconstructions with metal‐artifact reduction. The yellow rectangles indicate the ROIs in the artifact region and in a background region used to calculate the metal‐artifact metrices.

The phantoms and ROI variations are shown in Figure 2. Three different metal configurations were scanned for the electron‐density phantom: titanium (subfigures A and E), titanium and stainless steel (subfigures B and F), and titanium and two stainless steel rods (subfigures C and G). Additionally, a phantom was constructed from a water bath and hardware materials (screws and a bracket) to create very strong metal artifacts (subfigures D and H).

#### 2.4.4 Patient scans

**FIGURE 3:**
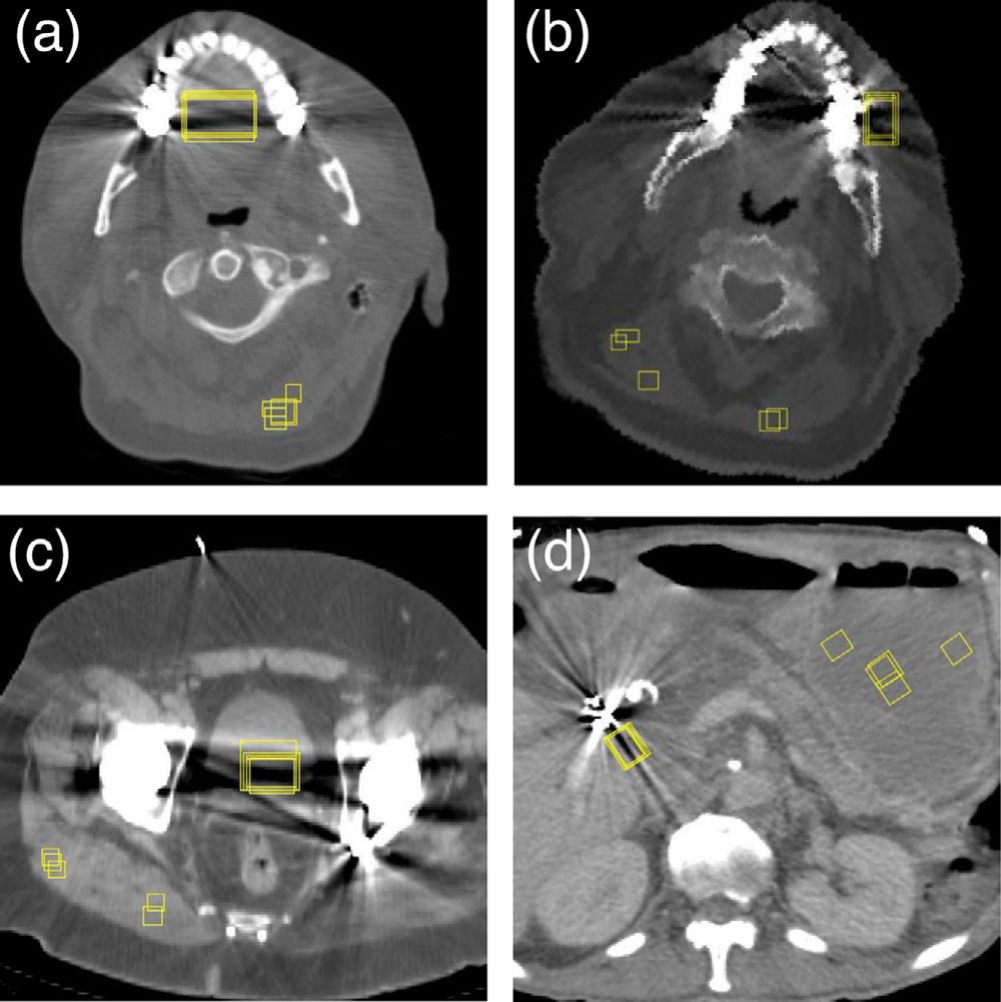
Four examples of metal artifacts in patient scans. (a), (b): artifacts from dental fillings, (c) artifacts from double‐hip implant, (d) artifacts from abdominal stent. The yellow rectangles indicate the ROIs in the artifact region and in a background region used to calculate the metal‐artifact metrices.

Four datasets^22^, ^23^, ^24^ were selected from the Cancer Imaging Archive^21^ containing patients with metal implants. Two scans had artifacts from dental implants, one from a double hip‐implant, and one from a stent (Figure 3).

#### 2.4.5 Setup reproducibility in phantom scans

The phantom in Figure 2(b) was set up on the CT table and scanned ten times. The ROIs were redrawn on each of the ten reconstructed scans. This procedure simulated repeated scans at different time points, with slight variations in the phantom position and ROI selections.

## 3 RESULTS

### 3.1 Quadratic sum rule of the Gumbel location parameter

**FIGURE 4:**
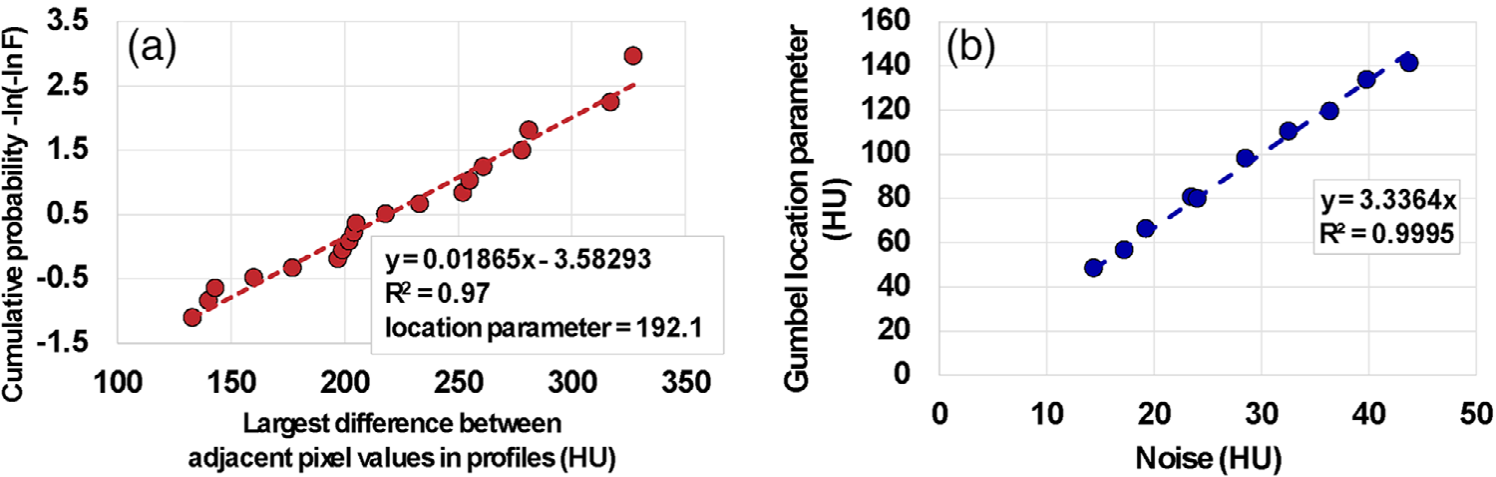
(a) Example of a Gumbel plot to determine the location parameter in a noise‐only image. (b) Fit of the location parameters against noise.

ROIs of 100 × 100 pixels were drawn at the center of the noise‐only images described previously. The Gumbel location parameter was calculated for each noise magnitude and plotted against the pixel standard deviation (Figure 4).

The example of the Gumble plot in Figure 4a indicates that the data points are linear and can be approximated by a straight line. The plot of the location parameters against the noise magnitude in Figure 4b also demonstrates a linear relationship, indicated by the high *R* ^2^ value of 0.9995 for the straight‐line fit without offset parameter.

In order to verify the quadratic sum rule of the Gumbel location parameter *λ*, pairs of noise images were added, and the measured location parameter was compared to the calculated value obtained from the quadratic sum of the location parameters of the original two images. The results in Table 1 show that the quadratic sum rule is valid for the Gumbel location parameter.

**TABLE 1:** Verification of the quadratic sum rule for the Gumbel location parameter *λ* in noise‐only images. SD is the standard deviation (noise).

SD 1 (HU)	SD 2 (HU)	λ 1 (HU)	λ 2 (HU)	Estimated λ for summed image: λ 1⊕λ 2 (HU)	Measured λ in summed image (HU)	Relative difference
43.8	36.4	141.4	119.5	185.1	184.4	0.4%
19.3	14.4	66.3	48.5	82.2	82.6	−0.6%
39.8	17.2	133.8	56.7	145.3	144.8	0.4%

### 3.2 Comparison of artifact metrics

The four artifact metrics were compared in terms of noise‐dependence, dependence on small variations in the choices of ROIs, and repeated setups (phantom scans only).

#### 3.2.1 Dependence on noise

**FIGURE 5:**
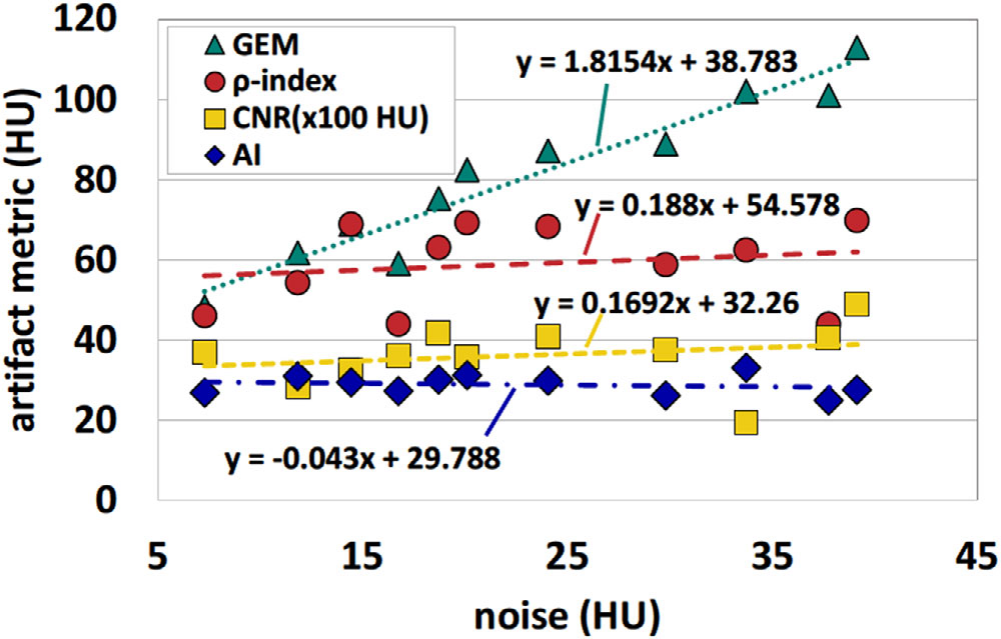
Noise dependence of the four metal artifact metrics. The CNR values were multiplied by 100 HU to fit into a similar data range as the other three metrics. Each dataset was fitted with a straight line and the parameters of the fitted curves a displayed next to each line.

The phantom with a titanium insert was scanned with an exposure of 800 mAs to obtain a low‐noise image, resulting in 7.3 HU of noise in the background ROI2. Ten additional noise magnitudes between 11 HU and 40 HU, measured in ROI2, were generated as described previously by adding noise‐only images. The plot of each artifact metric against the noise in Figure 5 shows that all but the GEM are noise‐independent over the range of simulated noise magnitudes. Straight‐line fits to the data yield only small slopes for CNR, AI, and ρ‐index. In contrast, the GEM shows a strong noise dependence, in agreement with the findings in the previous section.

#### 3.2.2 Dependence on the choice of the ROIs

This section summarizes the sensitivity of the four artifact metrices to small variations in the selection of the ROIs.

#### 3.2.3 Phantom scans

Coefficient‐of‐variation (COV) values over five ROIs for each of the phantom scans, both without and with MAR, are listed in Tables 2 and 3, respectively. The CNR shows the largest variation, with average values of 36.3% and 40.6% in the reconstructions without and with MAR, respectively. The COV for the AI is reduced compared to the CNR, yet remains substantial, with an average value of 12.6% for the MAR reconstructions. In contrast, both the GEM and ρ‐index demonstrate lower average COV values than the CNR and AI.

**TABLE 2:** Coefficients of variation in phantom scans without MAR. ‘Ti’ indicates a phantom with a titanium rod and ‘SS’ indicates a stainless steel rod.

Coefficient of variation	CNR	AI	GEM	ρ‐index
Repeated setups	48.8%	3.2%	2.5%	2.5%
Phantom Ti	64.7%	9.6%	2.9%	3.1%
Phantom Ti + SS	38.3%	8.4%	0.5%	0.5%
Phantom Ti + SS×2	17.3%	3.3%	4.3%	4.8%
Hardware phantom	12.5%	4.6%	6.2%	6.3%
Average	36.3%	5.8%	3.3%	3.4%

**TABLE 3:** Coefficients of variation in phantom scans with MAR. ‘Ti’ indicates a phantom with a titanium rod and ‘SS’ indicates a stainless steel rod.

Coefficient of variation	CNR	AI	GEM	ρ‐index
Repeated setups	30.0%	6.4%	6.7%	7.2%
Phantom Ti	31.7%	9.0%	5.5%	12.3%
Phantom Ti + SS	77.4%	14.1%	1.4%	3.7%
Phantom Ti, SS×2	49.6%	17.2%	4.2%	9.9%
Hardware phantom	14.5%	16.1%	6.5%	6.5%
Average	40.6%	12.6%	4.9%	7.9%

A detailed analysis of each ROI revealed that the AI is relatively robust under small shifts of the ROI (without size changes), but it is sensitive to the area encompassed by the ROI in the direction perpendicular to the artifact streaks. The AI resulted in slightly smaller COV values than GEM and ρ‐index for a few phantom configurations, but no consistent pattern emerged regarding which metric exhibits higher robustness compared to others. The CNR underperforms consistently compared to the other metrics with the hardware phantom with MAR reconstruction being the only exception.

#### 3.2.4 Patient scans

COV values over five ROIs for each patient scan are listed in Table 4. The GEM and ρ‐index result in lower COV values for all patient datasets compared to the AI. The CNR shows average COV values that are significantly higher than all the other metrics and similar to the ones observed in the phantom scans.

**TABLE 4:** Coefficients of variation in patient scans (without MAR).

Coefficient of variation	CNR	AI	GEM	*ρ*‐index
Patient (A)—dental fillings	59.4%	4.8%	1.6%	1.5%
Patient (B)—dental fillings	23.7%	12.8%	5.0%	5.0%
Patient (C)—hip implants	26.2%	17.3%	1.9%	2.0%
Patient (D)—stent	45.0%	4.5%	1.3%	1.3%
Average	38.6%	9.9%	2.5%	2.5%

#### 3.2.5 Setup reproducibility in phantom scans

Coefficients of variations for each of the metal artifact metrics for ten repeated setups are listed in Table 5. The CNR again demonstrates its sensitivity to minor variations of the ROI size and position relative to the artifact with a COV close to 50% without MAR and 30% with MAR. The other three artifact indices all show similar sensitivity to the phantom setup with moderate average COV values slightly below 5%.

**TABLE 5:** Coefficients of variation for the four artifact metrics for repeated phantom setups and scans.

Coefficients of variation	CNR	AI	GEM	*ρ*‐index
without MAR	48.8%	3.2%	2.5%	2.5%
with MAR	30.0%	6.4%	6.7%	7.2%
Average	39.4%	4.8%	4.6%	4.9%

## 4 DISCUSSION

Four metrics for quantifying metal artifacts in CT images (CNR, AI, GEM, *ρ*‐index) were investigated with respect to noise dependence and robustness in repeated measurements. The CNR was hypersensitive to minor changes in position, size, and pixel values of the chosen ROIs. It also did not correlate well with the strength of the artifact.^11^ Slightly different definitions for CNR can be found in the literature. In this work, the denominator was the quadratic difference between the pixel value standard deviation in ROI1 and ROI2 (“CNR1”). Other authors^25^ applied the quadratic sum instead of the difference (“CNR2”) or divided only by the ROI2 standard deviation^15^ (“CNR3”). Although not shown in this work, neither variation improved robustness of the CNR.

The AI quantifies the artifact by the standard deviation of pixel values. It is sensitive to the size of the ROI along the profile direction, and its value decreases for the same artifact strength the more unaffected area is included. In contrast, the GEM and ρ‐index are insensitive to the amount of “normal” area in the ROI because they measure the largest differential in pixel values which is generally dominated by the artifact itself. The noise dependence of the GEM can be mitigated by normalization to the Gumbel location parameter of a background ROI unaffected by the metal artifact, motivating the introduction of the ρ‐index.

Combining the results from Tables 2, 3, 4, 5, the *p*‐values for the hypothesis that the means of the coefficients of variation are equal between the *ρ*‐index and the other indices are $p\;=\;3\times 10^{-6}$ (CNR), *p *= 0.004 (AI), and *p *= 0.965 (GEM). Rejecting the null hypothesis for *p *< 0.05, these results demonstrate that the ρ‐index outperforms the CNR and AI, while it performs comparably to the GEM, as expected. It is important to note, though, that the *p*‐values are largely driven by the results from the patient data, and the AI resulted in a smaller COV in several of the phantom scans.

**FIGURE 6:**
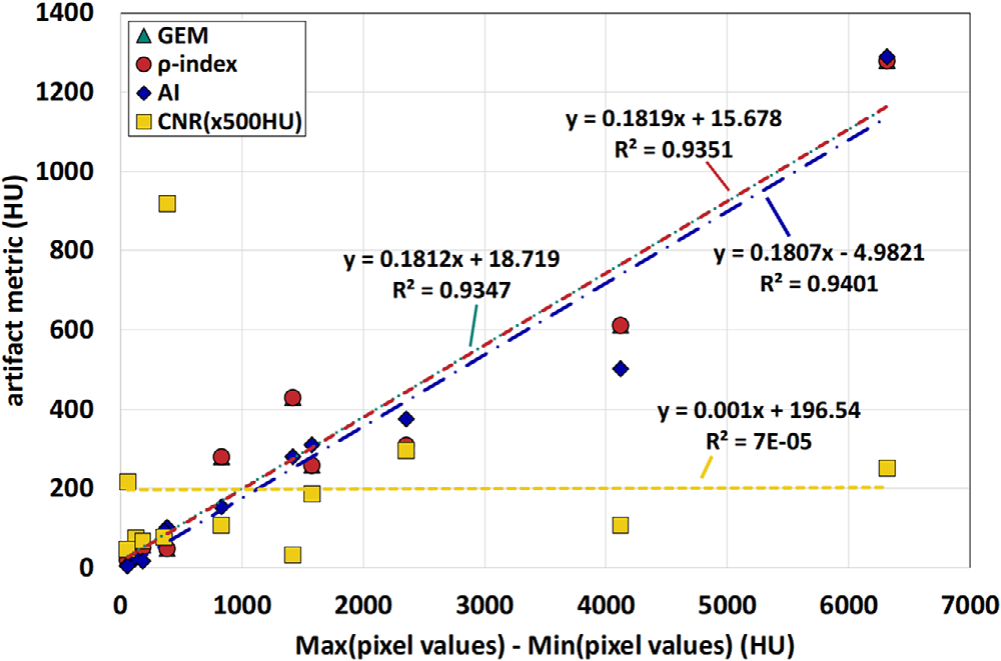
Correlation of the artifact metrics with the strength of the artifact, evaluated as the average of the range (maximum–minimum) of pixel values within the ROI1s for each phantom. Linear functions were fitted to each dataset. The data points for the GEM and the *ρ*‐index lie almost on top of each other.

A metric that quantifies artifacts should correlate with the artifact strength. While a comprehensive characterization of artifacts should also include their shape and size, the most prominent feature of metal artifacts are bright and dark streaks. Therefore, the difference between brightest and darkest pixel values (the pixel value range), averaged over all artifact ROIs drawn in each phantom or patient, was used as a simple metric to characterize the artifact strength. The artifact metrics are plotted against the artifact strength in Figure 6, along with linear fits to the data to assess their correlation. Both phantom scans with and without MAR were included, and the scans with MAR contributed most of the data points with smaller artifact strength, clustered in the lower left corner of the plot. The AI, GEM, and *ρ*‐index values exhibit a linear dependence on the artifact strength, as indicated by high values of the coefficient of determination (*R* ^2^), which are approximately 94%. In contrast, the linear fit to the CNR has slope and *R* ^2^ values close to zero, suggesting that the CNR metric does not correlate with the artifact strength.

Additional artifact metrics have been proposed or investigated in the literature beyond the metrics presented in this work. Große Hokamp^6^ defined circles and annular regions around a metal rod within which various artifact metrics were defined in both the spatial and frequency domains. They found higher correlation with observer rankings for the frequency‐domain method. Kim^26^ quantified metal artifacts in dental CBCT images by counting edges defined by a Canny edge detection algorithm. They concluded that the edge‐counting method is more robust than pixel value‐based methods due to larger gray‐level fluctuations in CBCT compared to CT. Chou^14^ compared four MAR algorithms from three vendors by assessing CT number accuracy and CNR defined by water and air inserts. They also evaluated the reconstructed area of the metal inserts against the true values and defined an artifact metric (“fraction of affected pixel area”, FAPA) by thresholding water from non‐water pixels. They found FAPA to be the most consistent among all investigated methods and concluded that the CNR “was not the ideal approach”, in agreement with the findings in this study.

The ρ‐index has been shown in this work to be noise‐independent and relatively robust to minor variations in the selection of the ROIs. It performed effectively in both phantom images (with and without MAR) and clinical images, exhibiting a strong correlation with the artifact strength. As such, it is a suitable metric for clinical applications. The ρ‐index is straightforward to derive from rectangular ROIs and can be calculated within the image domain with just a few lines of code or even within a spreadsheet software, making it a more robust and practical metric for quantifying metal artifacts than several other quantitative metrics proposed in the literature. Code implementations for the Python programming language and MATLAB (The MathWorks Inc., Natick, Massachusetts) are provided in the Supplementary material.

The ρ‐index has several limitations. Like other common metrics, it assesses the strength of the artifact by the difference in pixel values in bright and dark streaks but does not consider other properties such as the number of streaks or the artifact shape. The CNR (as defined in this study), AI, and *ρ*‐index all make use of a background ROI for noise‐normalization. If the fluctuations of pixel values in ROI1 and ROI2 are similar, for example, after applying a MAR algorithm, then two relatively similar numbers are subtracted in the normalization process, potentially leading to instabilities in the results. Additionally, some MAR algorithms have been observed to produce overly smooth interpolations of areas originally affected by metal artifacts, resulting in unnaturally low noise magnitudes. If the noise in ROI1 is less than the noise in the background ROI2, then the ρ‐index is ill‐defined, as are CNR and AI. In this case one could fall back to using the GEM as a robust artifact metric, albeit sacrificing the noise independence.

## 5 CONCLUSIONS

The ρ‐index has been proposed as a new metric for quantifying metal artifacts in CT images. It is independent of the underlying image noise and more robust under setup variations and the choice of the region‐of‐interest than several other metrics commonly found in the literature. The *ρ*‐index correlates strongly with the artifact strength and is easy to calculate without the need for complex image processing or mathematical transformations. This makes the ρ‐index well‐suited for repeated measurements, such as comparing artifact strength in periodic QA with phantoms, but also for assessing metal artifacts in clinical images.

## AUTHOR CONTRIBUTIONS

J. Cammin is the sole contributor to this manuscript and was responsible for the conception and study design, data collection, analysis, and interpretation, and for drafting and revising the manuscript.

## CONFLICT OF INTEREST STATEMENT

The author declares no conflicts of interest.

## Supporting information

Supporting Information
