# Supplementary material for: A robust index for metal artifact quantification in computed tomography
Source: J Appl Clin Med Phys. 2024 Jun 25;25(8):e14453. doi: 10.1002/acm2.14453 (PMC11302826; doi:10.1002/acm2.14453)
Supplement: Supplementary file 1 — Supporting Information [file ACM2-25-e14453-s001.zip › acm214453-sup-0001-SuppMat.doc]

# Supporting material for article “A robust index for metal artifact quantification in computed tomography”

# Creating an input text file with ROI pixel data

The pixel values in the ROI indicated in the Figure below are included in this supplement as an example (file “ROI.csv”).

For other images, the pixel values in a ROI can easily be saved as a CSV file using the free ImageJ software and the menu commands “Analyse / Tools / Save XY Coordinates” (see figure below). Other options include writing dedicated code to extract this information from the DICOM file.

Saved as CSV file:

X,Y,Value

205,120,51

206,120,93

207,120,142

208,120,102

209,120,123

210,120,114

211,120,84

212,120,38

…

# Calculating the Gumbel location parameter

The code examples included in the supplement (Python and MATLAB) read the data from a text file formatted as comma-separated values (CSV). The code calculates the maximum pixel value difference in each profile and plots the data against the estimated cumulative probability function of the Gumbel distribution. A linear fit to the data is then performed and the fit parameters are extracted to calculate the Gumbel location parameter. The output is shown below for Python code.

Python code example

```
import pandas as pd
import matplotlib.pyplot as plt
import numpy as np
import os, sys

os.chdir(sys.path[0]) # Change current directory to script directoy
df = pd.read_csv('ROI.csv') # Read the input CSV file

# Sort DataFrame by 'X' and 'Y' columns
sorted_df = df.sort_values(by=['X', 'Y'])

# Calculate parameters and values
nx = sorted_df['X'].max() - sorted_df['X'].min() + 1
ny = sorted_df['Y'].max() - sorted_df['Y'].min() + 1
data = sorted_df['Value'].to_numpy()

max_values = [np.max(np.abs(np.diff(data[i*ny:(i+1)*ny]))) for i in range(nx)]
F = [(i+1)/(nx+1) for i in range(nx)]
logsF = [-np.log(-np.log(prob)) for prob in F]
sorted_max_values = np.sort(max_values)

# Plotting
plt.figure()
plt.scatter( sorted_max_values, logsF, color='blue', alpha=0.6, label='Data' )
plt.xlabel('largest difference between adjacent pixel values (HU)')
plt.ylabel('-ln(-ln F)')
plt.grid(True)

# Linear fit
b, a = np.polyfit( sorted_max_values, logsF, deg=1)
xseq = np.linspace(sorted_max_values[0],sorted_max_values[-1],100)
plt.plot(xseq, a+b*xseq, color='b', ls='--', lw=2., alpha=1.0, label='Linear Fit')

# Display fit parameters and Gumbel location parameter inside the figure
fit_info = f'Fit parameters: a = {a:.4f}, b = {b:.4f}\
\nGumbel location parameter = {-a/b:.4f}'
plt.text(0.45, 0.07, fit_info, transform=plt.gca().transAxes, fontsize=10, bbox=dict(facecolor='white', alpha=0.8))

# Show legend and show plot
plt.legend(loc='upper left')
plt.tight_layout()  # Ensures tight layout to prevent overlap
plt.show()
```

# MATLAB code example

% Change working directory to the script's location

cd(fileparts(mfilename('fullpath')));

% Read the CSV file into a table

csv_data = readtable('ROI.csv');

% Sort the table by 'X' and 'Y' columns

sortedData = sortrows(csv_data, {'X', 'Y'});

% Calculate parameters and values

nx = max(sortedData.X) - min(sortedData.X) + 1;

ny = max(sortedData.Y) - min(sortedData.Y) + 1;

data = sortedData.Value;

maxValues = arrayfun(@(i) max(abs(diff(data((i-1)*ny + 1:i*ny)))), 1:nx);

F = (1:nx) / (nx + 1);

logsF = -log(-log(F));

sortedMaxValues = sort(maxValues);

% Plot data

figure;

scatter(sortedMaxValues, logsF, 'blue', 'filled', 'DisplayName', 'Data');

hold on;

% Linear fit

p = polyfit(sortedMaxValues, logsF, 1);

xseq = linspace(sortedMaxValues(1), sortedMaxValues(end), 100);

plot(xseq, polyval(p, xseq), '--b', 'LineWidth', 2, 'DisplayName', 'Linear Fit');

% Display fit parameters and Gumbel location parameter inside the figure

fitInfo = sprintf('Fit parameters: a = %.4f, b = %.4f\nGumbel location parameter = %.4f', p(2), p(1), -p(2)/p(1));

text(0.4, 0.07, fitInfo, 'Units', 'normalized', 'FontSize', 10, 'BackgroundColor', 'white', 'EdgeColor', 'black');

% Legend placement and plot labeling

legend('Location', 'northwest');

xlabel('largest difference between adjacent pixel values (HU)');

ylabel('-ln(-ln F)');

grid on;

hold off;
